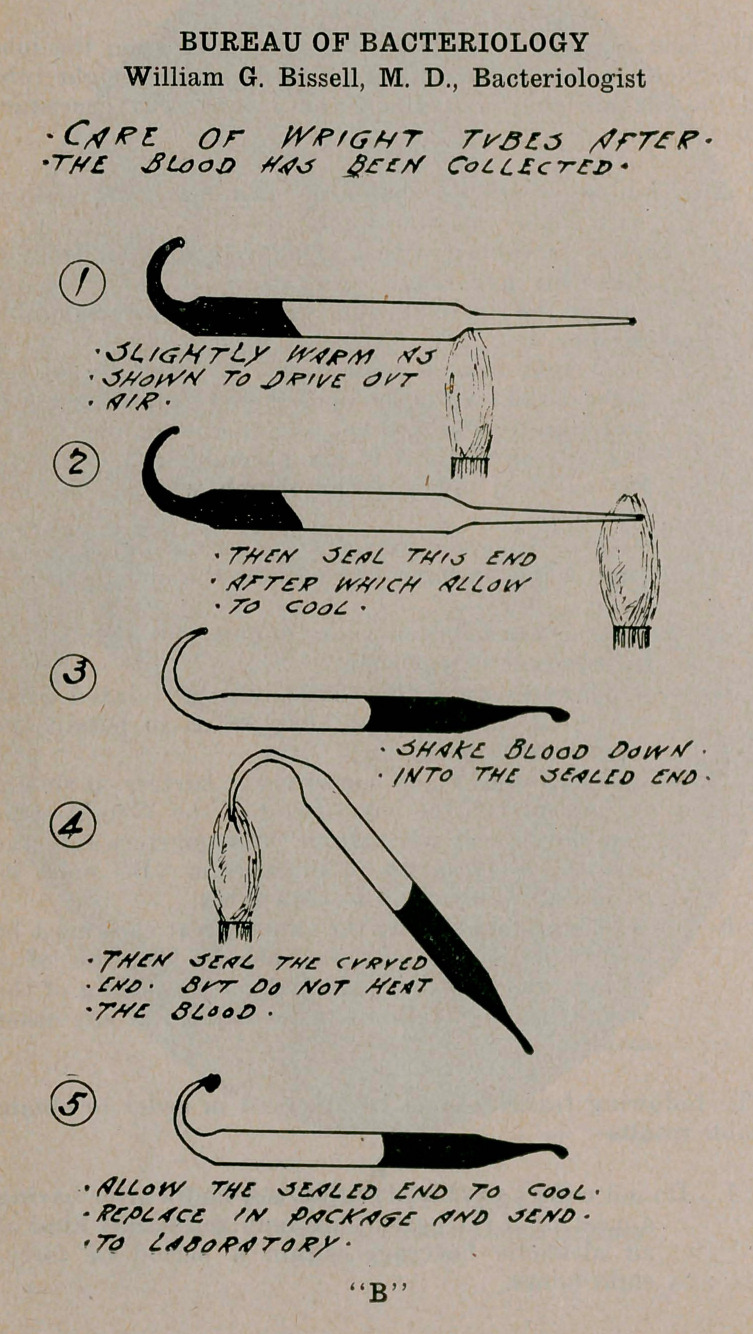# The Wasserman Test in the Municipal Laboratory and the Interpretation to Be Placed upon Results

**Published:** 1914-07

**Authors:** William G. Bissell

**Affiliations:** Bacteriologist. Chief of the Bureau of Bacteriology, Dept. of Health, Buffalo, N. Y. Major Medical Corps, N. G., N. Y., (retired) 1st Lieut. Medical Reserve Corps, U. S. A.


### "The Wasserman Test in the Municipal Laboratory and the interpretation to be placed upon results."

By WILLIAM G. BISSELL, M. D., Bacteriologist.

Chief of the Bureau of Bacteriology, Dept. of Health, Buffalo, N. Y. Major Medical Corps, N. G., N. Y., (retired) 1st Lieut. Medical Reserve Corps, U. S. A.

Read before the Pathological Section—Buffalo Academy of Medicine, May 19, 1914.

In presenting the subject under the title given it will be the effort of the writer to do so in a manner that will not only be of assistance to the general practitioner in understanding the limitations of this class of laboratory investigation, but also explain some of the difficulties that will unquestionably arise with other municipal laboratories contemplating an introduction of the test as a routine procedure.

As a rule city laboratories have little opportunity to observe the clinical pictures presented by those furnishing the material for tests and it is generally recognized that laboratory investigations to be of any great amount of value should be in accordance with clinical observations. With the complement fixation test for Syphilis, or as it has been popularly termed, "The Wassermann Test," many features present themselves which are most complicated in character, and as the test is not one of absolute specificity, the Laboratory results will frequently mislead the general practitioner, providing he is not thoroughly familiar with their interpretations. When it was definitely decided to introduce the test as a routine procedure in the work of the Laboratory of the Department of Health, Buffalo, N. Y., a most careful and thorough investigation was started to ascertain the features necessary to care for a large number of samples, using a system that would be practical in its application and reliable in results. The system originally adopted by Wassermann (4) was studied and compared with such modification as recommended by Noguchi (5) Hecht & Fleming (11) Simon (3) Citron (4) Meier (4) Porges & Meier (2) Landsteiner (4) (2) Muller & Potzi (4) Weidanz (4) and Craig (12) (8) (7). It was found that the method of Craig's was decidedly more adaptable for Municipal Laboratory use in that it WAS RELIABLE, and afforded advantages which the writer will endeavor to describe.

Before understanding a description of the "Wassermann Test," it will be necessary to explain the meaning of certain technical terms used to designate the ingredients employed and the reactions obtained.

First on the list is the word COMPLEMENT introduced by Ehrlich. It is a substance present in nearly all freshly drawn blood serum and has the peculiar property of gradually changing after the collection of the serum and can be completely destroyed (thermolabile) by heating the serum to a temperature between 55 and 56 degrees centigrade for one half hour. For laboratory purposes it has been found that complement contained in the serum from the blood of the guinea pig, and about 15 hours old, is the most serviceable.

The term AMBOCEPTOR likewise introduced by Ehrlich is another principal found in the serum of the blood of many animals and can be artificially produced in the serum of certain animals by the injecting of various substances. Amboceptor (Haemolytic Amboceptor) used in the "Wassermann Test" is produced artificially in the serum of an animal—usually the rabbit—by injecting washed blood corpuscles from another specie of animal blood. Repeated injections of these corpuscles develops the amboceptor properties in the serum of the rabbit so treated.

WASHED BLOOD CORPUSCLES. This is merely collecting definite qualities of blood in a solution of Citrate of Soda and the after sedimenting of the corpuscles by centrifugal force, repeatedly suspending the corpuscles in a normal salt solution and continued recentrifugation until all the serum from the blood has been removed. Washed blood corpuscles are not only used for the production of the amboceptor qualities in the serum of the rabbit, but also to determine the reaction known as HAEMOLYSIS.

HAEMOLYSIS. It has been known for a considerable period of time that if washed blood corpuscles, complement, and the amboceptor (produced by injecting the same variety of washed corpuscles as used in the mixture) be placed together in definite proportions in normal salt solution and incubated at the body temperature, that the corpuscles will become disintegrated, liberating the Haemoglobin contained in their composition and imparting the color to the salt solution. This process of disintegration of red blood cells, through the action of complement and amboceptor is known as Haemolysis, and its employment as a reactive feature in the "Wasserman Test" for Syphilis constitutes "The Haemolytic System" of the test. In general application there are several varieties of this system all named after the specie of animal from which the blood corpuscles are obtained, and the term "anti" prefixed to the name of the animal to designate the variety of the system. For example if sheep's blood corpuscles are employed and are used for injection into the rabbit the amboceptor obtained is anti-sheep amboceptor and the Haemolytic System is the anti-sheep system. Again, if human blood corpuscles are used to determine the haemolytic reaction they must be employed to produce the amboceptor, and the system is known as the anti-human Haemolytic system.

ANTIGEN. An Antigen is any substance capable of producing an immune body by its introduction into an animal. These immune bodies are called anti bodies, and the use of the term Antigen in the complement fixation test for Syphilis means a substance containing materials that when brought in contact with anti bodies in the serum from syphilitic sources will have a reactive influence.

As regards the origin of the complement fixation test, Bordet & Gengou (2) demonstrated that if the blood serum of an animal rendered immune by the injection of a certain substance, was added to a mixture of that certain substance, in the presence of complement, and then incubated at the body temperature, a definite change took place, as shown by the fact that if washed blood cells and amboceptor of the same Haemolytic System as the cells were added to the mixture, and this mixture reincubated, the blood corpuscles did not dissolve (Haemolysize), or in other words there was **inhibition of Haemolysis.** It is explained that on account of the specific nature contained in the blood serum tested to the antigen used, that the complement added, **is fixed,** and as free complement is always necessary to produce the dissolving of the blood corpuscles with the corresponding Amboceptor, Haemolysis does not occur. It is this principle in the Bordet & Gengou phenomena, that Wassermann employed in determining the presence or absence of syphilitic infection in human blood.

Before describing the system in use in the Laboratory of the Department of Health of Buffalo, it may be of interest to relate some of the incidents experienced during a process of evolution, in determining what system was best to employ. After becoming thoroughly familiar with the technique in the complement fixation test, the writer visited many of the larger laboratories of the country and was impressed with the great variance in methods used. In almost each instance the particular laboratory visited was either using an entirely different system from that originated by Wasserman or modifications in the system, and hardly any two laboratories were working exactly alike. Duplicate samples of blood submitted to the different laboratories most frequently gave widely different results. It was not until the writer became acquainted with the system in use by Craig & Nichols at the Army Medical School that any uniformity of results was observed. Permission was obtained to work in the Laboratory of the Army Medical School under the immediate direction of Craig and as a result it has been possible to adopt a system for Municipal Laboratories, which has not only proven reliable so far as our present knowledge of the "Wassermann Test," but capable of caring for large numbers of samples of blood.

As regards the collection of samples, the greatest care must be exercised in a Municipal System and a circular letter pertaining to this feature was mailed to the physicians of Buffalo. The letter read as follows:

"Beginning with the new year, the laboratory of the Health Department will perform the "Wassermann" blood test for the physicians of the City, free of charge, providing the person from whom the blood is collected resides within the City of Buffalo.

It must be remembered that this test, which is most complicated in character, depends upon many features for its reliability, aside from the skill with which it is conducted in the laboratory, and unless these features are observed, will furnish results misleading in their interpretation.

To secure satisfactory results, it is of the utmost importance that the blood furnished be collected with the greatest care, following absolutely the directions outlined in the circular accompanying each outfit. The outfit that will be used consists of two sterilized Wright's tubes enclosed in an envelope which in turn is encased in an outside covering containing a circular of directions and a card for record. The outfits can be obtained only at the Health Department and after use must be delivered to the same place.

In warming and sealing the tubes, the greatest care must be exercised not to heat the blood. In order to have the test of practical value to the physician, certain features must be observed.

It is absolutely essential that the use of a Bunsen gas burner is available, as it is difficult to properly seal the Wright tube in any other manner. The samples of blood should not be taken within two hours after the ingestion of a meal, or if the person has indulged in any kind of an alcoholic beverage within a period of forty-eight (48) hours. The surface of the ear must be perfectly clean to avoid unnecessary bacterial contamination and free from all moisture, as the presence of moisture renders the blood unsuitable for a reliable test. In cleaning the ear, antiseptics should not be used. After collecting the blood, the process of sealing the tubes must be carefully followed.

The Wasserman test will be performed Tuesday of each week after January 1st, 1914, and the results sent to the physicians by mail.

Used outfits must be received at the Health Department not later than Monday evening to insure their being included in the weekly test.

It must be remembered that the Wassermann Reaction is to be considered an accessory in aiding the diagnosis. There are diseases which give blood tests similar to that of Syphilis—among these being Yaws (90%) Leprosy (50%) Malaria (febrile stage% ?), Pityriasis rosea (few cases reported), Carcinoma (especially involving the nervous system), Scarlet Fever (eruptive stage % ?) and possibly some cases of Tuberculosis. Treatment also influences the reaction.

It can be understood that the practical value of the test depends as much upon the care with which the blood is collected and the interpretation placed upon results, as the actual procedures in the laboratory."

There are many features that the clinician must constantly keep in mind prominent among them being the fact that it has been unquestionably proven (13) that blood serum from undoubted syphilitic patients that are not undergoing any treatment will vary in its powers to react by the complement fixation test and this variance may range anywhere from a negative reaction to a strongly double plus within a period of a few days.

It must likewise be remembered that the blood of persons giving a negative result may react strongly double plus after receiving a treatment with salvarsan providing the case is one of syphilis. (7 page 28).

The circular of directions accompanying each outfit reads as follows:

**DIRECTIONS FOR COLLECTING BLOOD FOR THE WASSERMAN TEST**

**"A"**

**DEPARTMENT OF HEALTH**

**Francis E. Fronczak, Health Commissioner**

Buffalo, N. Y.

To secure satisfactory results, it is of the utmost importance that the blood furnished the laboratory be collected with the greatest care, following absolutely the directions outlined in this circular.

The best location for the blood collection is from the lobe of the right ear, and the manner of holding the Wright tube and the ear are shown in illustration "A." The procedure is as follows:

A. The lobe of the ear should be clean, carefully washed with ether and allowed to dry.
B. The ear is rubbed with a clean, dry towel until it is decidedly hyperemic.
C. Puncture the rim of the lobe with a fair size triangular needle.
D. The Wright tube is held nearly horizontally in the right hand, with the curved end toward the ear and slightly lower than the punctured lobe.
E. Three fingers of the left hand should be in front of the ear and opposite the thumb which is behind the lobe.
F. The ear is squeezed between the second finger of the right hand and the fingers and thumb of the left hand.
G. Press the ear between these fingers and thumb, but do not pull downward.
H. The pressure upon the ear should be relaxed after obtaining each drop of blood so as to permit the capillaries to refill.
I. As each drop of blood comes to the surface, it should be collected in the curved end of the Wright tube.
J. When the tube is one-half to three-quarters full, the procedures as shown in illustration "B" must be carefully followed. Use both tubes.
K. In warming and sealing the tubes, great care must be exercised not to heat the blood. The use of a Bunsen gas burner is essential, as the sealing of the Wright tubes is difficult to accomplish in any other manner.

The following features must be observed in order to obtain reliable results:

1. Do not collect the blood within two hours after having a meal, or if the patient has indulged in any kind of an alcoholic beverage within a period of forty-eight hours.

2. The surface of the ear must be perfectly clean and free from all moisture. In cleaning the ear, do not use antiseptics.

**BUREAU OF BACTERIOLOGY**

**William G. Bissell, M. D., Bacteriologist**

"B"

3. After collecting the blood, be sure to seal the tubes, as shown in illustration "B," and send them as soon as convenient direct to the Health Department.

The Wasserman test will be performed Tuesday of each week and the results mailed as soon as possible. The used outfits must be received at the Health Department not later than Monday evening of each week to insure their being included in the test. The blanks accompanying the outfits must be completely filled out.

When the samples are received at the Laboratory the tubes containing the blood are centrifugated and placed in a paraffine bath between 55 and 56 degrees centigrade for thirty minutes. The object of these measures is to obtain a clear serum, and to destroy (inactivate) the presence of any possible complement that might be contained in the human blood serum. The test then proceeds as follows: Test tube racks having a double row of holes are used for the reception of specially designed small test tubes and there is placed in each tube nine tenths of a C. C. of steril normal salt solution. In both rows of tubes there is placed four capillary drops of the serum to be tested, and the mixture is thoroughly shaken. There is placed in both rows of tubes two units of complement, and in the front row, one unit of the syphilitic antigen. The mixtures are again thoroughly shaken and incubated in a water bath at a temperature of 37 degrees centigrade, for thirty minutes. All tubes are reshaken once during this period of incubation. The racks holding the tubes are then removed and there is added to each tube one tenth of a C. C. of a ten per cent. suspension of washed human red corpuscles in steril normal salt solution and two units of an anti-human amboceptor. The mixtures in all tubes are again shaken and reincubated, this time for a period of one hour, shaking during intervals of every fifteen minutes. At the completion of this period the racks containing the tubes are placed in an ice box and the results read after three hours exposure to the cooler temperature.

The designations used in the Health Department Laboratory of Buffalo are those suggested by Noguchi (5) and adopted by Craig (12) and as as follows:

**Double Plus** meaning complete inhibition of Haemolysis.

**Plus** meaning that fifty per cent. of the red blood corpuscles have been disintergrated with a corresponding color resulting in the fluid.

**Plus Minus** meaning any grade of further disintergration of red blood corpuscles and color up to that of **Minus** which is a complete disintergration of the corpuscles and a blood red shade.

Before considering the significance to be attached to the various grades of reactions the methods by which the Syphilitic Antigen, the Complement and Haemolytic Amboceptor are obtained will be given.

### Syphilitic Antigen

A variety of methods have been thoroughly studied and the writer believes that antigens made from the tissues from syphilitic sources are more stable and are preferable to any others yet suggested. The method used at the present time is that recommended by Walker & Swift (10) with the exception that the antigenic extract is obtained from the hearts of persons dying of Paresis. (It being considered that Paresis is due to Syphilis.) In other respects the method as published in the Journal of Experimental Medicine Volume XVIII No. 1 is followed. Antigens remain active much longer when kept at the room temperature. In the titrations of the antigens the methods as recommended by Craig (12) are adhered to. These methods, in addition to determining the actual antigenic strength, show whether the antigen in itself is haemolytic or contains anti-complementary properties, or both. The tables for titration are as follows:

### Titration of Antigen for Haemolytic Properties

(The antigen is diluted by adding 1 part to 9 parts normal salt solution.)

| Tube No. | Amount of salt solution. | Units of complement (50% dilution) | Amount of antigen emulsion 1 to 9 dil. | Amount of blood suspension 10% |
|---|---|---|---|---|
| 1 | 0.9 c.c. | 2 units | 0.05 c.c. | 0.1 c.c. |
| 2 | 0.9 c.c. | 2 units | 0.10 c.c. | 0.1 c.c. |
| 3 | 0.9 c.c. | 2 units | 0.15 c.c. | 0.1 c.c. |
| 4 | 0.9 c.c. | 2 units | 0.20 c.c. | 0.1 c.c. |
| 5 | 0.9 c.c. | 2 units | None | 0.1 c.c. |

Incubate in water bath at 37 degrees C for one hour, shaking every fifteen minutes.

None of the tubes show haemolysis. If haemolysis is present in all, including Tube No. 5 (the control tube without antigen) it would indicate that haemolytic substances are present in the complement serum, or the blood suspension, or both. If, however, haemolysis occurs only in one or more of the tubes containing the antigen emulsion, the control remaining unhaemolysed, it demonstrates that the antigen is haemolytic and, in this event it should be discarded.

The determination of the presence or absence of anti complementary properties are given in the following table:

(Dilute Antigen the same as in Haemolytic Titration)

| Tube No. | Amount of salt solution. | Units of complement. | Amount of Antigen emulsion. | Amount of blood suspension. | Units of Amboceptor. |
|---|---|---|---|---|---|
| 1 | 0.9 c.c. | 2 units | 0.05 c.c. | 0.1 c.c. | 2 units |
| 2 | 0.9 c.c. | 2 units | 0.1 c.c. | 0.1 c.c. | 2 units |
| 3 | 0.9 c.c. | 2 units | 0.15 c.c. | 0.1 c.c. | 2 units |
| 4 | 0.9 c.c. | 2 units | 0.2 c.c. | 0.1 c.c. | 2 units |
| 5 | 0.9 c.c. | 2 units | None | 0.1 c.c. | 2 units |

Incubate in water bath at 37 degrees C for one hour, shaking every fifteen minutes.

As a result of this titration there should be complete haemolysis in all of the tubes. If the control tube No. 5 shows inhibition of haemolysis, it will demonstrate that something is wrong with either the complement, amboceptor, or blood suspension, but if this No. 5 shows haemolysis, and any of the tubes containing the antigen emulsion show inhibition of haemolysis, it demonstrates that the antigen is anti complementary and under such circumstances it should be discarded.

The antigenic strength of the antigen is obtained by titrating it with a known positive serum in the following manner:

**Titration of Antigen to Determine Antigenic Properties.**

(Dilute Antigen same as in Haemolytic Titration)

| Tube No. | Amount of salt solution. | Amount of syphilitic serum (inactivated) | Amount of complement. | Amount Antigen emulsion (1 to 9) | Amount of blood suspension 10% | Amount of Amboceptor. |
|---|---|---|---|---|---|---|
| 1 | 0.9 c.c. | 4 drops (.08 c.c.) | 2 units | 0.05 c.c. | 0.1 c.c. | 2 units |
| 2 | 0.9 c.c. | 4 drops (.08 c.c.) | 2 units | 0.1 c.c. | 0.1 c.c. | 2 units |
| 3 | 0.9 c.c. | 4 drops (.08 c.c.) | 2 units | 0.15 c.c. | 0.1 c.c. | 2 units |
| 4 | 0.9 c.c. | 4 drops (.08 c.c.) | 2 units | 0.2 c.c. | 0.1 c.c. | 2 units |
| 5 | 0.9 c.c. | 4 drops (.08 c.c.) | 2 units | None | 0.1 c.c. | 2 units |
| 6 | 0.9 c.c. | Normal Serum 4 drops (.08 c.c.) | 2 units | 0.1 c.c. | 0.1 c.c. | 2 units |
| 7 | 0.9 c.c. | Normal Serum 4 drops (.08 c.c.) | 2 units | 0.2 c.c. | 0.1 c.c. | 2 units |
| 8 | 0.9 c.c. | Normal Serum 4 drops (.08 c.c.) | 2 units | None | 0.1 c.c. | 2 units |

(After antigen:) Incubate in water bath at 37 degrees C for half an hour—Shake once.

(After amboceptor:) Incubate in water bath at 37 degrees C for one hour, shaking every 15 minutes.

As a result of this titration all the tubes containing antigen and syphilitic serum should show absolute **inhibition of haemolysis.** Control tube 6 containing normal serum and 0.1 c.c. of antigen and control tube 7, containing normal serum and 0.2 c.c. of the antigen, should show **complete haemolysis, while** control tube 8, containing normal serum and no antigen should also show **complete haemolysis.** Control tube 5 containing syphilitic serum with no antigen should show **complete haemolysis.**

If these results are obtained, the titration has demonstrated that the antigenic emulsion used is capable of producing inhibition of haemolysis in a dose as small as 0.05 c.c. and that it does not inhibit haemolysis in the presence of normal serum even in four times that dose. Therefore the antigen is a suitable one to use in complete fixation tests.

An antigenic unit may be defined as the smallest amount of antigenic emulsion that will produce absolute inhibition of haemolysis in 1 c.c. of a 1 per cent. suspension of erythrocytes in the presence of two units each of complement and amboceptor in the presence of 4 drops of syphilitic serum, and this is the amount that is used in Craig's method for the complement fixation tests for syphilis.

As regards the **Complement** that obtained from guinea pigs has been demonstrated to be the most desirable. Well nourished and fully grown pigs are bled by severing the carotid artery, allowing the blood to flow directly into a sterilized petri dish. The dish containing the blood is permitted to remain at the room temperature for about two hours, after which time it is placed in an ice box. The most convenient procedure is to bleed the animal late in the afternoon of the day preceding its use allowing the blood to remain in the ice box for a period of from 12 to 15 hours. The blood will thoroughly clot during this time and the clear serum can be poured off in a most convenient manner. Experiences show that serum collected by this manner is most suitable for use. In using the serum it is the practice in the Health Department's Laboratory to dilute it with equal parts of normal salt solution. In performing the "Wasserman Test," and the use of complement for that purpose, Craig (7) very emphatically insists that each lot of complement should be titrated each time before use as different lots of complement not only materially differ in their original strength, but will diminish in its powers with age beyond certain limitations. This procedure is always followed:

The method of titrating complement is as follows:

(Dilute the serum with equal parts of normal salt solution)

| Tube No. | Amount of salt solution. | Amount of blood suspension. 10% in normal salt solution. | Amount of amboceptor. | Amount of complement. |
|---|---|---|---|---|
| 1 | 0.9 c.c. | 0.1 c.c. | 1 unit | 0.02 c.c. |
| 2 | 0.9 c.c. | 0.1 c.c. | 1 unit | 0.04 c.c. |
| 3 | 0.9 c.c. | 0.1 c.c. | 1 unit | 0.05 c.c. |
| 4 | 0.9 c.c. | 0.1 c.c. | 1 unit | 0.06 c.c. |
| 5 | 0.9 c.c. | 0.1 c.c. | 1 unit | 0.07 c.c. |
| 6 | 0.9 c.c. | 0.1 c.c. | 1 unit | 0.08 c.c. |
| 7 | 0.9 c.c. | 0.1 c.c. | 1 unit | 0.10 c.c. |
| 8 | 0.9 c.c. | 0.1 c.c. | None | 0.10 c.c. |

Incubate in water bath at 37 degree C. for one hour, shaking every fifteen minutes, and then read result.

After incubating in the water bath at 37 degrees C. for one hour, the smallest amount of serum that has caused haemolysis is noted, and this constitutes one unit of complement. For example, if it is found that the tube containing 0.05 c.c. of complement (Tube No. 3) shows complete haemolysis, and the tubes containing less are only partly haemolysed, then 0.05 c.c. of the serum, diluted in the proportion stated, constitutes one complement unit, and in making the Wasserman test twice this amount or 0.1 c.c. of the diluted complement is used for each tube. The control tube No. 8 should show no haemolysis.

**A Complement Unit** is the smallest amount of complement that will cause complete haemolysis of 1 c.c. of a one per cent. suspension of erythrocytes in the presence of one unit of amboceptor in 60 minutes at 37 degrees C.

**The production of Haemolytic Amboceptor.** The haemolytic system used in the Department of Health Laboratory of Buffalo is that suggested by Noguchi (6) and adopted by Craig (12) known as the anti-human. With this system the employment of human red blood corpuscles is necessary and an amboceptor produced by the injection of washed human blood corpuscles is employed. Experience has demonstrated that large quantities of human blood suitable for this work can be readily obtained from the Lying-in Institutions in large cities. It is the writer's custom to furnish small flasks containing definite quantities of a sterilized two per cent. solution of citrate of soda, the flask being marked so that definite quantities of blood may be collected from the placental end of the severed cord during the time of parturition. Blood collected in this manner has been found to be sufficiently steril. The blood is distributed in large centrifugal tubes and the corpuscles deposited after which normal salt solution is added and the mixture recentrifugated, the washing process being repeated until all albumen has been removed, as shown by the nitric acid test of the clear fluid.. The method for animal immunization is that suggested by Noguchi (5) using gradually increased amounts of red blood corpuscles starting with 5 c.c. and increasing until 25 c.c. is the amount used, allowing a period of from 5 to 7 days between injections and injecting the corpuscles intraperitoneal after removing the hair from the animal and staining the outer skin with iodine. After nine days following the last injection, the animal is bled from the carotid artery into a large petri dish and the blood is allowed to coagulate by remaining at the room temperature at least two hours after which it is placed in an ice box. As soon as a clear serum presents itself it is incorporated upon filter paper and quickly dried under a current of air from an electric fan. The determination of the amboceptor unit is as follows:

**Preliminary Titration of Amboceptor Paper**

| Tube No. | Amount of salt solution. | Amount of complement. | Amount of blood suspension 10% | Amount of Amboceptor paper 3 . M.M wide |
|---|---|---|---|---|
| 1 | 0.9 c.c. | 1 unit | 0.1 c.c. | 1 M.M. |
| 2 | 0.9 c.c. | 1 unit | 0.1 c.c. | 2 M.M. |
| 3 | 0.9 c.c. | 1 unit | 0.1 c.c. | 3 M.M. |
| 4 | 0.9 c.c. | 1 unit | 0.1 c.c. | 4 M.M. |
| 5 | 0.9 c.c. | 1 unit | 0.1 c.c. | 5 M.M. |
| 6 | 0.9 c.c. | 1 unit | 0.1 c.c. | None |

Incubate in water bath at 37 degrees C. for one hour.

After incubating in a water bath at 37 degrees C. for one hour, the titration is read, and if any of the tubes containing from one to five M.M. of the paper is completely haemolysed, the paper is strong enough for practical purposes. There should be no haemolysis in Tube No. 6.

It is the writer's experience that any method of conducting the "Wasserman Test" by the use of anti sheep system is open to very serious error, for the reason that human serum oftentimes has an amboceptor or haemolysin, a property itself of sheep's corpuscles, and the determination of units necessary to employ in the test is most difficult. Human beings undoubtedly are to a degree immunized against sheep tissues probably through the agency of lamb and mutton being used for human food, and for this reason any method using an anti human system seems preferable. The only objection to the anti human system is the inconvenience and sometimes difficulty in procuring sufficient quantities of human blood, but with Municipal Laboratories in large cities the writer finds that this objection can be easily overcome.

If an anti sheep haemolytic system, such as outlined in the original test by Wasserman, must be employed, the procedure, described by Simon (3) of diluting the inactivated human serum to be tested with 5 volumes of the sheep corpuscular emulsion, and allowing this mixture to incubate thirty minutes with subsequent centrifugation to obtain a clear fluid for testing should be employed. Failure to carry out some procedure that will remove the normal sheep amboceptor in human sera is undoubtedly one reason why so many workers using the original Wasserman technique with anti sheep haemolytic system have so many varied results and as Simon (3) has so admirably stated "The Objectionable Nachlosung." This procedure of depletion adds an additional complication to an already overburdened test and the necessity for its use can be entirely eliminated by the use of the anti human system, suggested by Noguchi (5), as applied by Craig (12).

The significance to be attached to the various grades of reaction, in the writer's opinion, cannot be more appropriately expressed than quoting practically verbatim from a portion of an article by Charles F. Craig, published in Bulletin No. 3 of the Surgeon General's Office, U. S. A.: Dr. Craig (7) states:

"I have been impressed with the uncertainty that appears to exist among the profession regarding the interpretation to be placed upon the results obtained with the complement fixation test for syphilis, especially as regards **diagnosis** and the **control of treatment.** Many patients have been told that they were free from syphilis upon the strength of a single negative reaction, while the diagnosis of syphilis has been repeatedly made, in my experience, on the presence of a plus-minus or a plus reaction in the absence of a history of infection and any symptom of the disease. Such interpretations of the reaction are entirely unwarranted and have led to the infection of innocent individuals, on the one hand, and to great mental and physical suffering by those unjustly stigmatized as infected with this disease on the other.

In any consideration of this phase of the subject, a clear conception must be had of the meaning of the terms employed in reporting reactions. In the Army Laboratory there is used four designations for the reaction, **double-plus, meaning a positive reaction; plus and plus minus, doubtful reactions**; and **minus a negative reaction.** It is necessary that these terms and their meaning be kept clearly in mind in order to understand the discussion that follows:

The significance to be attached to the various grades of reaction reported varies with the stage of syphilis in which they are obtained. Thus, a plus reaction in the primary stage of syphilis means much more than it does in the secondary stage, as a considerable proportion of primary cases never give more than a plus reaction before the development of secondary symptoms. Therefore it will be necessary to consider the interpretation of the reaction for each stage of the disease.

**Interpretation of the results in the primary stage of syphilis**: In this stage of the disease a diagnosis should only be based upon a plus or a double-plus reaction. While the plus-minus reaction, especially if obtained during the first and second weeks after the appearance of the suspicious lesion, is of some value, it is best to consider such reactions as negative and have the test repeated within a week or two, when, if the disease is syphilis, a plus or a double plus reaction will probably be obtained.

A double plus reaction in the primary stage of syphilis is absolutely diagnostic, provided the few conditions in which such a reaction is occasionally given can be excluded. It will be noted that in 13.8 per cent. of primary cases tested during the first week a double-plus reaction was obtained and that this percentage increased until by the fiftieth week 61 per cent. of the cases gave a double-plus reaction. It will be seen that a very considerable proportion of cases in the primary stage give a double-plus Wasserman test.

It is my (Craig's) belief, that a plus reaction in the primary stage of syphilis, should be given nearly as much value as a double plus, but under the same limitations. Craig has never observed a case of primary syphilis giving a plus reaction that afterward became negative, unless as the result of treatment. If there is a clear history of infection, or a suspicious lesion is present, a plus reaction should be regarded as a positive reaction, provided more than one test be made with the same result. In the absence of either a history of infection, or a lesion, a plus reaction should not be considered as diagnostic.

Craig has already stated that a plus-minus reaction should not be considered as diagnostic of syphilis in the primary stage of the disease and that repeated examinations should always be made in such cases.

**The significance of a negative reaction during the primary stage of syphilis is practically nil.** Between 10 and 20 per cent. of cases in this stage of the disease give a negative reaction, and it therefore follows that even repeated negative reactions in this stage do not prove the absence of syphilis.

**Interpretation of the results in the secondary stage of syphilis.** Of 1,582 cases of syphilis in the secondary stage of the disease, 1,518 or 95.9 per cent., gave a positive reaction. In those giving a negative reaction secondary symptoms were present in all, so that it must be admitted that between 4 and 5 per cent. of secondary cases give a negative result.

A double-plus reaction in patients presenting suspicious symptoms of this stage of syphilis is conclusive evidence of the presence of the disease under the limitations already mentioned in discussing the interpretation of the test during the primary stage.

A plus reaction when symptoms are present or when there is a clear history of a primary lesion, while not of as great value in this stage as in the primary is practically conclusive evidence of syphilis. A plus reaction is observed in about 15 per cent. of cases in the secondary stage of syphilis, even when very active symptoms are present, so that a very careful consideration of all the features of the case must be given before one ignores the presence of a plus reaction in the secondary stage.

A plus-minus reaction in this stage of syphilis is valueless from a diagnostic standpoint and one does a great injustice to the patient if a diagnosis of syphilis is made on such a reaction. In Craig's experience, either a double-plus or a plus reaction is invariably obtained in cases that react at all, unless treatment has been given. When this is the case, the treatment should be omitted, the test repeated after an interval of two or three months, and if the reaction is still plus-minus, the diagnosis of syphilis should not be made in the absence of other symptoms. However, if the reaction has become plus or double-plus a diagnosis of syphilis may be made.

A negative reaction in supposed cases of secondary syphilis, is of greater diagnostic value than in any other stage, but even here **we must not forget that practically 5 per cent of secondary cases presenting active symptoms give a negative reaction and this reaction may persist upon repeated examinations.** While this is true, a negative reaction in the supposed secondary stage of syphilis, persisting over several months, and in the absence of further symptoms of the disease may be regarded as conclusive, provided no treatment has been administered. The same interpretation may be made of a negative reaction on those cases having an indefinite or no history of infection and no typical symptoms provided the negative result is obtained upon repeated examinations.

**Interpretation of the results in the tertiary stage of syphilis.** The Army data show that between 12 and 15 per cent. of cases in the tertiary stage of syphilis give a negative reaction, a fact of great importance in interpreting the result of the test during this stage of the disease.

A double-plus reaction in the tertiary stage of syphilis is diagnostic under the limitations already noted.

A plus reaction occurs in a larger proportion of the tertiary than of the secondary cases and is therefore of greater diagnostic value. In patients presenting suspicious tertiary lesions, Craig believes it should have the same value as a double-plus reaction, provided it is repeatedly plus and with the same limitations that have been noted for this grade of reaction in the other stages of syphilis.

A plus-minus reaction is of no diagnostic value in the tertiary stage unless it is permanent or treatment has been administered. Repeated examinations should always be made in such cases before the condition is diagnosed as syphilis, but in the presence of a clear history and of symptoms a plus-minus reaction should not lead one to consider the case as non-syphilitic.

A negative reaction in patients suspected of tertiary syphilis is only of value in excluding the disease when it is repeatedly negative over a period of many months and typical symptoms have not developed. As Craig's results show that practically 15 per cent. of cases of tertiary syphilis, presenting symptoms, give a negative reaction, it is evident that this alone is not sufficient to enable one to be sure that the patient is free from the disease, unless the test is repeated as stated. In the absence of typical symptoms and a history of infection a negative reaction, if repeatedly negative may be interpreted as indicating the absence of syphilis, provided no treatment has been administered.

**Interpretation of the results in latent syphilis.** Of patients tested in the latent stage of syphilis, 65.2 per cent., gave a positive reaction. Almost all of these cases had received more or less treatment and none of them presented symptoms of the disease beyond glandular enlargements. It is in this class of cases that the value of the Wasserman test in the diagnosis of syphilis, is best illustrated, but one must be very careful in interpreting anything but a double-plus reaction as diagnostic of the disease.

A double-plus reaction is not obtained as often in the latent stage as in the active stages of syphilis, but when obtained is diagnostic under the limitations already mentioned.

The interpretation of a plus reaction offers the greatest difficulty in this class of cases, as we obtain more plus reactions in latent syphilis, than double plus reactions. A plus reaction, provided there is a clear history of infection, and if specific treatment has been administered for some time, should have the same value in diagnosis as a double-plus reaction. Craig believes that in such cases a plus reaction is diagnostic of syphilis under the limitations already noted. When there is no history of infection a diagnosis of the disease should not be made on a plus reaction.

As most patients applying for a Wasserman test in the absence of symptoms have been treated for the disease at an earlier period, and are anxious to ascertain whether they are cured, a greater value has to be given, in such instances, to the plus-minus reaction. If there is a history of infection and treatment has been administered, a plus-minus reaction should arouse the suspicion that the disease is still present and indicates the resumption of treatment, but two or three tests should be made before treatment is resumed. When there is no history of infection a plus-minus reaction is of no diagnostic significance.

A negative reaction in cases suspected of being in the latent stage of syphilis is of no value in excluding the disease, unless it is consistently negative over a long period of time. Statistics show that 35 per cent of latent cases gave a negative result and a considerable proportion of these later developed symptoms and the Wasserman became positive. It is therefore evident that a negative reaction does not exclude syphilis in cases suspected of being in the latent stage of the disease.''

In conclusion I wish again to quote directly the remarks of Dr. Craig on Page 44 of the Bulletin—Studies of Syphilis.

''If the disease in which the Wasserman complement fixation test has occasionally been found positive can be excluded (such as named in the circular sent to the Physicians of Buffalo) a double-plus reaction is sufficient to enable one to diagnose the presence of syphilis. **Under such conditions Craig considers the test absolutely specific, whether symptoms of the disease are present or not, and whether there is or is not a history of infection.**

Under the same conditions, and with a history of infection or the presence of clinical symptoms, a plus reaction should also be interpreted as diagnostic of syphilis.

A diagnosis of syphilis should never be made on the presence of a plus-minus reaction alone. Many normal individuals will give a plus-minus reaction at times, and therefore such a reaction cannot be considered as having any more value than a negative reaction in the absence of a history or symptoms.

**A single negative reaction is of no value in excluding syphilis.** Only when such a reaction is obtained on repeated examinations extending over at least a year can it be considered as good evidence of the disease. In the interpretation of a negative result, the history of the patient, the presence or absence of symptoms, and the amount of previous specific treatment must all be carefully considered.

**The interpretation of the results of the complement fixation test must, therefore, always rest with the clinician.** The laboratory simply reports the result of the test, without reference to data regarding the history of the case or the symptoms present, and it must rest with the clinician to correlate the laboratory report with the clinical condition present, and this is only possible when the clinician possesses a clear conception of the limitations of the "Wasserman Test" and of the interpretation which should be given the reaction in the various stages of syphilis."

### References

1. **Webster**—Diagnostic Methods—3rd Edition.
2. **Bolduan**—Immune Sera.
3. **Simon**—Infection and Immunity, 1913.
4. **Citron-Garbat**—Immunity—3rd Edition.
5. **Noguchi**—Serum Diagnosis of Syphilis.
6. **Panton**—Clinical Pathology.
7. **War Department, Office of Surgeon General**—Bulletin No. 3—Studies of Syphilis by Craig & Nichols.
8. **Charles F. Craig**—The Journal of the A. M. A. Volume LX, page 565.
9. **Charles F. Craig**—The Journal of the A. M. A. Volume LXII, page 1232.
10. **Walker & Swift**—The Journal of Experimental Medicine, Volume XVIII, page 75.
11. **Fleming**—London Lancet, 1909. 4474.
12. **Craig**—Notes taken during personal instruction at Army Medical School by writer.
13. **Charles F. Craig**—The Journal of the A. M. A., Volume LXII, page 1232.

---

**A Contribution to Our Knowledge of the Excretion of Phosphates in Infancy.** Knox and Tracy, in the American Journal of Diseases of Children, June, 1914, think that every infant admitted to the hospital should be placed on the metabolism bed for at least 24 hours to get an accurate knowledge of the kidney function. In their investigation they include 19 infants with 3 deaths. The determination of the phosphorus in the urine was made by the uranium titration method, the Neubauer method for the food mixtures, for the nitrogen determination the Kjelgahl-Gunning method . The results confirmed the observations of others that the urinary phosphorus secreted by artificially fed infants is greater that that recorded for the breast-fed child, but no deduction as to the nature or severity of the nutritional derangement can be drawn from the amount of urinary phosphorus.—C. G. L-W.

## Figures and Tables

**Figure f1:**
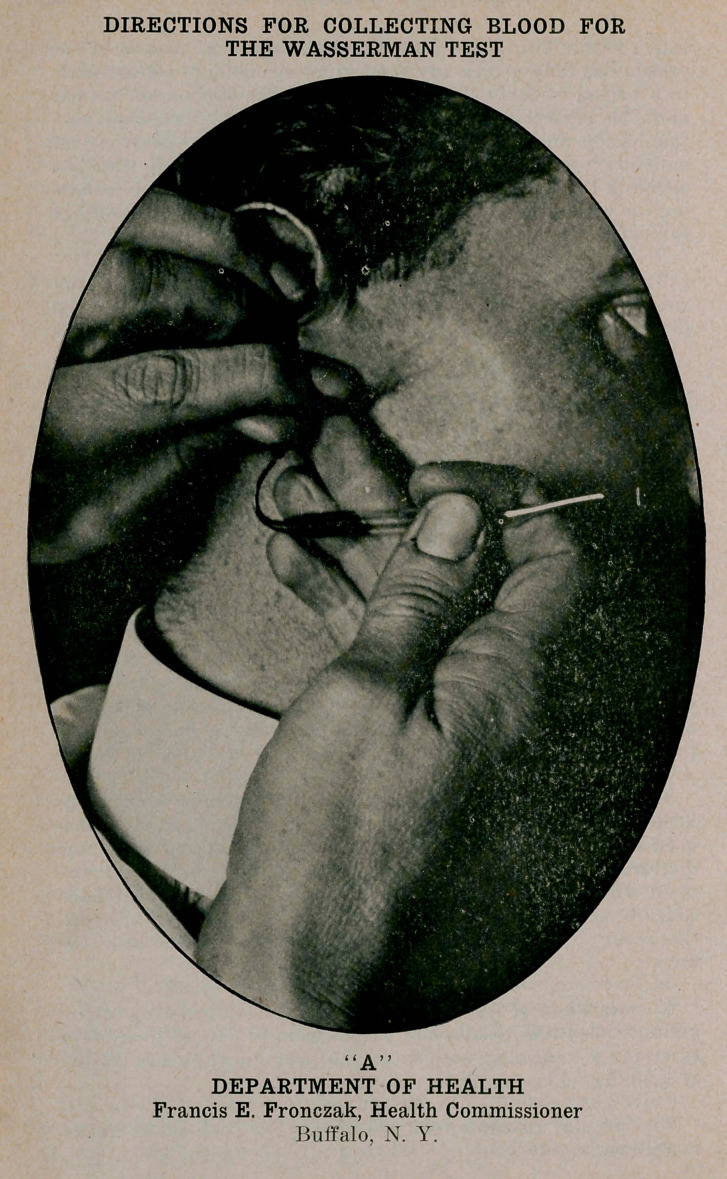


**Figure f2:**